# Predictors of Adherence to Antiretroviral Therapy among People Living with HIV in Northern Egypt

**DOI:** 10.5334/aogh.4491

**Published:** 2024-09-16

**Authors:** Mona Magdy, Adel Zaki, Sherif Omar Osman, Ekram W. Abd El-Wahab, Asmaa Abd Elhameed

**Affiliations:** 1 Department of Biomedical Informatics and Medical Statistics, Medical Research Institute, Alexandria University, 21561 Alexandria, Egypt; 2 Tropical Health Department, High Institute of Public Health, Alexandria University, 21561 Alexandria, Egypt

**Keywords:** Antiretroviral therapy, adherence, viral suppression, risk factors, barriers, predictors

## Abstract

*Background:* Adherence to medications is a crucial factor in achieving the best therapeutic outcomes for patients who have human immunodeficiency virus (HIV). Little is known about the rate and predictors of adherence to antiretroviral therapy (ART) in Egypt.

*Objectives:* To assess the degree of adherence to ART among people living with HIV/AIDS (PLWHA) in Egypt and to explore the predictors of non-adherence.

*Methods:* A cross-sectional study was conducted from January 2021 to December 2021 on 785 PLWHA attending an ART clinic at the main fever hospital in Alexandria, Egypt. Data collection was done using an interviewing questionnaire and pharmacy database records. Multivariate logistic regression analysis was done to identify the predictors of adherence to ART.

*Results:* The overall adherence rate to ART among the study subjects was 66.7%. Female sex (Adjusted Odds Ratio [95% CI]: 1.73 [1.01–2.96]), intravenous drug use (AOR [95% CI]: 2.87 [1.27–6.49]), fair satisfaction with the health service at ART clinics (OR [95% CI]: 1.86 [1.27–2.73]) appeared as independent predictors of poor adherence.

*Conclusion:* The degree of adherence to ART among PLWHA in Egypt is noticeably high, although it was influenced by several patient-, healthcare-, and community-related factors. This work provides an accurate, standardized tool to measure adherence and identify factors that contribute to non-adherence.

## Introduction

The number of people living with HIV/AIDS (PLWHA) worldwide continues to grow. According to the Joint United Nations Programme on HIV/AIDS (UNAIDS) global statistics, 39.0 million (33.1 million–45.7 million) people globally were living with HIV in 2022 [1]. Egypt is considered, by international standards, one of the lowest HIV-prevalence countries, with a prevalence in 2020 of less than 0.1% [2]. The World Health Organization (WHO) estimated the number of PLWHA in Egypt in 2021 to be 30.000 (28.000–34.000), whereas the reported number of those on antiretroviral therapy (ART) was 12.311, with a percentage of coverage reaching 45% [3]. The Egyptian Ministry of Health and Population (MoHP) declared in 2022 an annual increase in the number of newly identified HIV infections among adults by 20 to 25% [4].

Advancements in HIV treatment regimens have significantly improved adherence to medication. An adherence to ART of 95% is considered an appropriate level to achieve maximal viral suppression [5]. Antiretroviral therapy has been proven effective in achieving a good virological response, preventing viral transmission, and enhancing immune function while preventing drug resistance [6]. Consequently, ART has transformed HIV infection from a life-threatening condition to a manageable, chronic disease comparable to other common chronic illnesses like diabetes and hypertension [7]. Poor adherence to treatment, however, can lead to various unhealthy factors that lead to the development of permanent treatment resistance to specific HIV drugs or drug combinations. This, in turn, may result in increased treatment expenses and limited therapeutic choices for the patient [8].

Various factors can contribute to poor adherence to ART. These factors are diverse and encompass several aspects, such as the complexity of treatment regimens (e.g., the number of pills and dosing frequency), treatment side effects, limited health literacy, and weak patient–physician relationships. Socioeconomic factors such as low income and lack of education can also play a role in non-adherence. Furthermore, HIV-related stigma is a significant factor that affects adherence to ART and can impact the overall quality of life of PLWHA [9–12].

The WHO typically classifies measures of drug adherence as either subjective or objective metrics. However, each metric has benefits and drawbacks, and therefore they should be combined [13]. Subjective measurements are those that call for a physician or a patient to assess how well he or she is taking their medications, according to their self-report. The most-often-used instrument to measure drug adherence are assessments conducted by healthcare professionals. Objective measures include pill counts, electronic monitoring, secondary database analysis, and biological assays such as plasma viral load and CD4 count. Accordingly, it is important to validate and correlate the subjective metrics using the objective metrics [14]. Pharmacy database records can also be used to measure adherence. This database is checked when prescriptions are initially filled, refilled (each time), and even if the prescription is prematurely discontinued. However, this method requires the abilities to keep records and to follow patients over time [15, 16].

Across all US regions, PLWHA had adherence rates of 80–90% [17]. In some low-resource settings, the mean adherence levels can vary between 60% and 95% [18, 19].

In Egypt, a free ART program was initially launched by the MoHP in 2005 [2]. However, little is known about the rate and predictors of adherence to ART in Egypt so far. In 2006, Kabbash and co-workers conducted a study on 153 PLWHA from three cities (Cairo, Alexandria, and Gharbia governorates) with the highest number of reported cases of HIV in Egypt. The study identified some psychosocial and healthcare needs of PLWHA, but it did not address the barriers to adherence to HIV medication [20].

Therefore, the present work aimed to assess the adherence rate to ART among HIV patients in Alexandria, Egypt, and to explore predictors of non-adherence.

## Methods

### Study design, setting, and population

**Figure 1:**
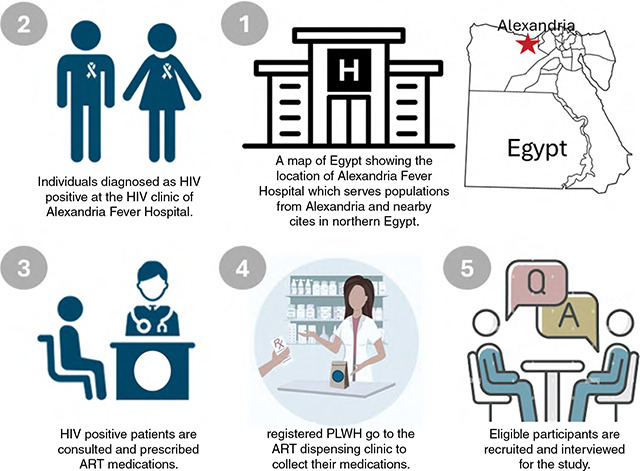
Recruitment of the study participants.

A cross-sectional study was conducted between January and December 2021 at the main Fever Hospital in Alexandria governorate, a city in the north of Egypt. Alexandria Fever Hospital is a pooling hospital that serves populations from Alexandria and nearby governorates. The target population was PLWHA attending the HIV clinic at Alexandria Fever Hospital, which is centralized for dispensing ART. Potentially eligible participants were defined as adults aged 18 years or older who were mentally well (not diagnosed with any mental disorder and demonstrating resilience and cognitive clarity), on ART as of 31 December 2020, and voluntarily willing to participate and sign an informed written consent. Those who were mentally ill or incarcerated (being inaccessible to the researcher) and those who were HIV-positive cases but unaware of their health status (which was undisclosed by the healthcare team) were excluded from the study (Figure 1).

Before proceeding to the main study, a pilot was conducted on 40 PLWHA to assess the feasibility of the study, to test the coherence of the questionnaire, and to provide the necessary information for calculating the sample size. Some questions were rephrased after the pilot study to ensure better clarity and achieve a high level of completion. Data from the pilot study were not included in the final study analysis to ensure both validity and reliability.

The sample size was calculated by the Power Analysis and Sample Size (PASS 12) program [21] to provide the minimum number of participants required to maintain an adequate precision in the study. We based the sample size calculation on an adherence level to ART of 86.0%, as revealed in the pilot study. It was based on an adherence level to ART of 86.0%, as revealed in the pilot study. Using a confidence interval (CI) of 95.0%, a degree of precision equal to 2.0%, and a level of significance set at 0.05, the calculated sample size was equal to 559. However, we eventually enrolled 785 PLWHA to compensate for any loss for follow-up and to improve the statistical power of the final analysis. Eight participants did not complete the study, and the final analysis was done on a sample of 777 PLWHA.

All HIV-positive adults (above 18 years of age) who were currently on ART follow-up at the HIV clinic of Alexandria Fever Hospital were invited to participate in the study. Those who agreed to participate signed an informed written consent and were given a more detailed description of the study’s aims and procedures. PLWHA were excluded from the study if they were mentally ill or incarcerated, and HIV-positive patients who were as yet unaware of their health status were also excluded.

## Data Collection

A face-to-face interview questionnaire was designed as the primary data-collection tool. The questionnaire was initially constructed in Arabic for ease of understanding and distributed in a printed form to the participants. Each interview took around 10–15 min to complete. The questionnaire consisted mostly of closed-ended questions but also included a few open-ended inquiries to provide more diverse details. The first part of the questionnaire asked for sociodemographic data (age, sex, marital status, residence, education, employment) as well as information on HIV-risk behaviors.

The second part of the questionnaire covered data on baseline ART adherence, follow-up, and the patient’s satisfaction with the health service.

### Assessment of adherence to ART

We assessed patients’ adherence to ART using the database records of the pharmacy dispensing ART at Alexandria Fever Hospital. The records were accurate and registered patients’ monthly visits, which we tracked over 12 months, starting from January 2021 to December 2021.

According to the National AIDS Program (NAP) in Egypt [22], a good level of adherence is achieved when the patient seeks care within six days (i.e., three days before or three days after the scheduled appointment) to receive his or her monthly treatment. Patients with an 80% or greater adherence level were considered to have good adherence [2]. Accordingly, adherence is defined as good if it is ≥80% and poor if it is <80%.

### Assessment of stigma among PLWHA

The 40-item “HIV Stigma Scale” covers all stigma processes affecting individuals with HIV and is the most widely used of the various tools meant to measure HIV stigma [23]. We assessed stigma among the enrolled participants using the short, 12-item version of the HIV stigma scale [24]. It consists of four sections that measure Personal stigma, Disclosure concerns, Concerns about public attitudes, and Negative self-image. Data on stigma among the enrolled PLWHA in this study were reported in an earlier publication (Magdy et al., 2023) [25].

### Statistical analysis

Collected data were checked for integrity and completeness, coded, and fed to computer software. Data were then analyzed using SPSS version ver. 22. Descriptive statistics were generated as frequencies and as percentages for categorical variables. Fisher’s exact test and bivariate logistic regression analysis (unadjusted odds ratio) were used to examine associations between levels of adherence and some independent variables. We then conducted a multivariate logistic regression analysis (adjusted odds ratios [AOR]) and 95% confidence intervals (95% CI)] to identify predictors that are independently associated with adherence to ART. In our multivariate model, we included all variables based on prior literature and biological plausibility, regardless of their statistical significance in the univariate analysis, using the “enter” method. In all statistical tests, a *p*-value of <0.1 was used as a set of statistical significance.

## Results

### Sociodemographic and clinical characteristics

A total of 785 adult PLWHA currently on ART follow-up were eventually enrolled in the study. Of the total participants, 567 (72.2%) were male, and 309 (39.4%) were married. The mean age of the study participants was 37.37 ± 10 years, while 67.8% of them were between the ages of 30 and 50 years. The majority (69.4%) of the study participants had less than 12 years of education.

### Adherence to ART among PLWHA

Most participants (99.8%) were on a “Once a day” pill regimen. Considering 80% as the cutoff point to determine adherence to ART, the data revealed that the majority of the participants (66.7%) achieved good adherence. On the other hand, 259 individuals (33.3%) fell below the 80% cutoff level, indicating poor adherence.

### Barriers to adherence to ART

In the bivariate analysis, residency, education, and HIV-risk behaviors were negatively associated with adherence to ART. Participants were also more likely to achieve poor adherence if they were residing in Alexandria (*p* = 0.040), of low literacy (*p* = 0.005), or intravenous drug users (IVDUs) (*p* < 0.001) (see Table 1).

**Table 1:** Association between patient-related factors and adherence to ART in PLWHA.

PREDICTORS	UNADJUSTED	*P-*VALUE	ADJUSTED	*P-*VALUE
	OR (95% CI)		OR (95% CI)	
**Sex**				
Male	Ref		Ref	
Female	1.142 (0.822–1.588)	0.429	2.023 (1.107–3.698)	**0.022**
**HIV-risky behavior**				
Blood transfusion	Ref		Ref	
Intravenous drug users	3.607 (1.656–7.853)	**0.001**	2.861 (1.216–6.732)	**0.016**
Heterosexuality	1.584 (0.782–3.207)	**0.202**	1.388 (0.645–2.986)	**0.402**
MSM	1.337 (0.600–2.980)	0.478	1.282 (0.513–3.097)	0.58
Unknown risk/ other risks	1.183 (0.535–2.617)	0.678	1.083 (0.473–2.480)	0.851
**Patient satisfaction with the care provided at the HIV clinic**				
Satisfied	Ref		Ref	
Unsatisfied	1.860 (1.315–2.630)	**< 0.001**	1.970 (1.274–3.048)	**0.002**
**Education**				
University or higher	Ref		Ref	
Illiteracy, read and write	2.196 (1.325–3.640)	**0.002**	1.850 (1.030–3.323)	**0.04**
≤12 years of education	1.531 (1.085–2.160)	0.015	1.337 (0.913–1.957)	**0.136**
**Employment**				
Student or employee	Ref		Ref	
Unemployed	1.905 (1.128–3.218)	**0.016**	1.677 (0.920–3.058)	**0.092**
Owner	1.328 (0.847–2.080)	0.216	1.407 (0.875–2.261)	0.159
Housewife or retired	1.009 (0.699–1.455)	0.963	0.729 (0.404–1.314)	0.293

~80% is the cutoff level of “Good adherence.”

ART: antiretroviral therapy

MSM = Men who have sex with men

OR: odds ratio

Ref: reference

*p* < 0.1 is set as a level of significance.

Dissatisfaction with clinic services was among the health-related factors that negatively affected the respondents’ adherence to the ART. PLWHA who expressed dissatisfaction with clinic services had greater likelihood of poor adherence (*p* < 0.001) (see Table 2).

**Table 2:** Association between healthcare-related factors and adherence to ART in PLWHA.

HEALTHCARE-RELATED FACTORS	GOOD ADHERENCE	POOR ADHERENCE	UNADJUSTED	*P-* VALUE
	*N* (%)	*N* (%)	OR (95% CI)	
**Duration of treatment**				0.182
2–4 years	286 (69.6)	125 (30.4)	Ref	
<2 years	63 (62.4)	38 (37.6)	1.38 (0.88–2.17)	
>4 years	169 (63.8)	96 (36.2)	1.30 (0.91–1.80)	
**Number of tablets**				0.575
> 2	61 (69.3)	27 (30.7)	Ref	
2	457 (66.3)	232 (33.7)	1.15 (0.71–1.85)	
**Patient satisfaction with the care provided at the clinic**				**< 0.001**
Satisfied	422 (69.9)	182 (30.1)	Ref	
Unsatisfied	96 (55.5)	77 (44.5)	1.86 (1.32–2.63)	
**Patient satisfaction with pharmacist service**				0.067
Satisfied	512 (67.1)	251 (32.9)	Ref	
Neutral	6 (42.9)	8 (57.1)	2.72 (0.93–7.92)	

~ 80% is the cutoff level of “Good adherence.”

ART: antiretroviral therapy

OR: odds ratio

Ref: reference

*p* < 0.1 is set as a level of significance.

We examined the association between adherence to ART and the four subscales of the stigma scale in this study. The subscale “*Negative self–image*” was significantly associated with good adherence to ART (*p* = 0.082), more particularly, question number 23 (“I feel guilty because I have HIV”) (*p* = 0.052) (see Table 3).

**Table 3:** Adherence to ART in relation to stigma subscales.

SCORES OF THE SUBSCALE	GOOD ADHERENCE	POOR ADHERENCE	*P-*VALUE
	*N* (%)	*N* (%)	
**1–Personal stigma**			0.616 ^a^
P1– Some people avoid touching me when they find out I have HIV.			
– Doesn’t happen/Rare	209 (63.0)	123 (37.0)	0.790 ^b^
– Sometimes/Always	51 (64.6)	28 (35.4)	
P2– People I care about stop calling after knowing that I have HIV.			
– Doesn’t happen/Rare	221 (62.3)	134 (37.7)	0.286 ^b^
– Sometimes/Always	39 (69.6)	17 (30.4)	
P3–I lost my friends because I told them that I have HIV.			
– Doesn’t happen/Rare	222 (62.5)	133 (37.5)	0.348 ^b^
– Sometimes/Always	38 (69.1)	17 (30.9)	
**2–Disclosure concerns**			0.343 ^a^
D1–Telling anyone that I have HIV is a risk.			
– Doesn’t happen/Rare	5 (45.5)	6 (54.5)	0.194 ^b^
– Sometimes/Always	512 (67.0)	252 (33.0)	
D2–I work hard to keep my HIV a secret.			
– Doesn’t happen/Rare	5 (50.0)	1 (50.0)	0.314 ^b^
– Sometimes/Always	513 (66.9)	254 (33.1)	
D3– I am very careful about telling anyone I have HIV (work).			
– Doesn’t happen/Rare	1 (50.0)	1 (50.0)	0.547 ^b^
– Sometimes/Always	374 (67.4)	181 (32.6)	
**3–Concerns about public attitudes**			0.477 ^a^
C1–People with HIV are treated like outcasts.			
– Doesn’t happen/Rare	8 (72.7)	3 (27.3)	0.759 ^b^
– Sometimes/Always	502 (66.2)	256 (33.8)	
C2–Most people believe that a person who has HIV is a badly behaving person.			
– Doesn’t happen/Rare	3 (60.0)	2 (40.0)	1.000 ^b^
– Sometimes/Always	514 (66.7)	257 (33.3)	
C3–Most people are not comfortable around someone who has HIV.			
– Doesn’t happen/Rare	10 (76.9)	3 (23.1)	0.559 ^b^
– Sometimes/Always	500 (66.1)	256 (33.9)	
**4–Negative self–image**			
N1–I feel guilty because I have HIV.			**–0.082 ^a^**
– Doesn’t happen/Rare	240 (70.4)	101 (29.6)	**0.052 ^b^**
– Sometimes/Always	278 (63.8)	158 (36.2)	
N2–People’s attitudes toward HIV make me feel worse about myself.			
– Doesn’t happen/Rare	83 (63.4)	48 (36.6)	0.339 ^b^
– Sometimes/Always	423 (67.7)	202 (32.3)	
N3–I feel that I am not as good as others because I have HIV.			
– Doesn’t happen/Rare	99 (62.7)	59 (37.3)	0.247 ^b^
– Sometimes/Always	416 (67.5)	200 (32.5)	

^a^: by Mann–Whitney U test

^b^: by Pearson Chi-Square test

~ 80% is the cutoff level of “Good adherence.”

ART; antiretroviral therapy

*p* < 0.1 is set as a level of significance.

Several factors appeared in our multivariate logistic regression model as predictors of adherence to ART. Female sex (AOR [95% CI] = 2.02 [1.11–3.70]), IVDUs (AOR [95% CI]= 2.86 [1.216–6.732]), dissatisfaction with clinic services (AOR [95% CI] = 1.97 [1.27–3.05]), low literacy (AOR [95% CI] = 1.850 [1.03–3.32]), and unemployment (AOR [95% CI] = 1.68 [0.92–3.06]) were associated with higher odds of having poor adherence to ART (Table 4).

**Table 4:** Predictors of poor adherence to ART in PLWHA.

PREDICTORS	UNADJUSTED	*P-*VALUE	ADJUSTED	*P-*VALUE
	OR (95% CI)		OR (95% CI)	
**Sex**				
Male	Ref		Ref	
Female	1.142 (0.822–1.588)	0.429	2.023 (1.107–3.698)	0.022
**HIV-risky behavior**				
Blood transfusion	Ref		Ref	
Intravenous drug users	3.607 (1.656–7.853)	0.001	2.861 (1.216–6.732)	0.016
Heterosexuality	1.584 (0.782–3.207)	0.202	1.388 (0.645–2.986)	0.402
MSM	1.337 (0.600–2.980)	0.478	1.282 (0.513–3.097)	0.58
Unknown risk/other risks	1.183 (0.535–2.617)	0.678	1.083 (0.473–2.480)	0.851
**Patient satisfaction with the care provided at the HIV clinic**				
Satisfied	Ref		Ref	
Unsatisfied	1.860 (1.315–2.630)	**<** 0.001	1.970 (1.274–3.048)	0.002
**Education**				
University or higher	Ref		Ref	
Illiteracy, read and write	2.196 (1.325–3.640)	0.002	1.850 (1.030–3.323)	0.04
≤12 years of education	1.531 (1.085–2.160)	0.015	1.337 (0.913–1.957)	0.136
**Employment**				
Student or employee	Ref		Ref	
Unemployed	1.905 (1.128–3.218)	0.016	1.677 (0.920–3.058)	0.092
Owner	1.328 (0.847–2.080)	0.216	1.407 (0.875–2.261)	0.159
Housewife or retired	1.009 (0.699–1.455)	0.963	0.729 (0.404–1.314)	0.293

– Variables initially included in multivariate logistic regression using the Enter method were age category, sex, marital status, potential HIV mode of transmission, number of tablets, duration of treatment, satisfaction with the clinic, satisfaction with the pharmacy, education level, residence, median score of the total stigma score, question no. 23, and employment.

– 10.2% of the variance of bad adherence to ART is explained by significant independent variables included in the model (Nagelkerke *R*^2^ = 0.102);

Model Summary: *X*^2^ =59.414, *p* < 0.001;

Hosmer–Lemeshow: *X*^2^ = 9.751, *p* = 0.283

~80% is the cutoff level of “Good adherence.”

ART: antiretroviral therapy

OR: odds ratio

Ref: reference

*p* < 0.1 is set as a level of significance.

## Discussion

This study assessed PLWHA’s adherence levels to ART and the associated factors at the HIV clinic of Alexandria Fever Hospital as a pooling center for measuring HIV cases from cities in Northern Egypt.

A high adherence rate was found among 66.7% of the participants, with an adherence rate of 80%. However, this rate is below the recommended level of ART adherence (≥ 95%) to adequately suppress viral replication, stop the disease progression, and bring the patient to clinical improvement [26]. Meanwhile, our data are concordant with the fact that adherence to ART has gotten much easier over time with newer ART combinations, particularly because once–daily regimens are easier to adhere to than are multi-dose regimens [27]. Our findings were also consistent with the many studies showing that people with chronic illnesses who take 80% of their prescribed medications are considered to be adherent to their therapy [28–30]. The adherence level found in the present study was higher than the mean adherence rate reported in meta-analysis studies (60–70%) [31, 32] but lower than the adherence level found among PLWHA in some regional countries (Ethiopia [83.0%–90.8%] and Nigeria [90.0%]) [33–36]. The variation seen in adherence levels could be attributed to many correlates, including different socioeconomic or demographic differences or different tools used to assess adherence levels. Researchers and healthcare providers should thus work toward establishing an operational, standardized definition of medication adherence that is recognized internationally in order to compare and reproduce medication-adherence findings. An accurate description of the medication-adherence techniques used, confirming the standard validity and reliability of the various measuring tools, should be considered as well.

Regarding patient-related factors, we found that the age, sex, education, and employment status of the respondents, as well as their HIV-risky behaviors, were important factors linked to ART adherence. The sex and age distributions of our study population were similar to those reported in other studies [37–39]. Indeed, males were more likely to be adherent to the treatment than were female participants. Similar male predominances have been found in other studies [38, 40]. Women who tested positive for HIV were more vulnerable to experiencing delayed care, with fewer possible visits to health centers, limited access to treatment, greater stigma, and poorer health outcomes [41]. These barriers may reduce adherence to ART, thus leading to lower rates of viral suppression.

Having heterosexual transmission as a possible HIV risk factor was the most common transmission-risk category among 53.1% of the participants. A similar finding was published in a study conducted in Oman among 1,427 HIV patients, where 66.3% of PLWHA reported heterosexuality as their possible HIV-transmission risk factor [37]. Compared to blood transfusion as a possible cause of HIV exposure, IVDUs and mainstream media (MSM) were more likely to show poor adherence to ART. These findings were similar to those of studies conducted in Oman [37], the United States [42], and Morocco [39]. and can be attributed to HIV-related stigma and stress, as blood transfusion is more morally acceptable than other modes of transmission. After some time has passed, the care of PLWHA who adhere poorly to ART will be focused on how to get them their drug dose with absolutely no adherence to ART [43].

It was also noted in our study that 114 patients (14.5%) reported having an unknown exposure source of HIV and did not disclose an actual mode of transmission. It is possible that they opted not to disclose such information for fear of stigma and criminal prosecution [44].

In the present study, we found that unemployed participants had poor adherence to ART compared to those who were employed. This observation was similar to that of a study revealing that the likelihood of virologic failure was twice as high when one was unemployed [45]. It was also consistent with studies reporting that PLWHA who had jobs were more likely to be more adherent and even achieved faster viral suppression [38, 46]. Having a fixed job and a fixed source of income makes patients feel secure and encourages them to be more adherent. Furthermore, our data showed that participants with higher education levels were more likely to adhere to the treatment. These findings were also consistent with a study done in Ethiopia conducted on 329 PLWHA, finding that the chances of adhering among those who were not able to read and write were roughly 90.0% less than those of patients with higher educational levels. Additionally, patients with low education levels had lower adherence compared to their higher-education-level counterparts [35], which possibly may be due to their having higher health-related knowledge and a better understanding of the disease’s nature [38, 47].

When we explored the patient–healthcare provider relationship, we found that patient satisfaction with physician-provided services in the clinic was a significant predictor of adherence to ART. A similar finding was obtained in a systematic review that included 15 studies and that looked for correlates and predictors of ART adherence among 4,363 participants across 10 low- and middle-income countries and found that low healthcare-mutual relations are associated with low adherence levels [48]. This was also similar to studies that found that a good relationship between HIV patients and healthcare providers affects adherence levels. In fact, instructions from the physician concerning medications and the importance of adhering to ART were consistently found to be crucial elements in patients’ adherence to HIV medication [49–51].

In conclusion, the adherence level among PLWHA in this study was considerably high. Adherence to ART was influenced by a variety of patient-related factors, healthcare provider–related factors, and cultural-related factors. The findings from this study are essential for developing strategies to improve adherence among PLWHA in Egypt.

Future research should examine the psychosocial impact of HIV stigma on health outcomes. A national strategy should be implemented to raise public awareness and to promote social support for PLWHA.

### Limitations and strengths

This study is not without limitations. First, we were unable to assess adherence using other objective measures such as laboratory tests to measure viral load or CD4 level to determine the extent of viral suppression and clinical improvement. Second, the data of participants who were lost to follow-up or who died might have underestimated their adherence level. Eighteen patients died during the study period; however, we excluded only eight patients (1.0%) because they died at the start of the study, whereas the data from the other 10 patients were eventually usable. Third, we lacked potentially beneficial data on other patient-related factors, such as comorbidities and mental health, that probably could have had an impact on the adherence level. Moreover, all patients were currently receiving care at one center. However, the Alexandria Fever Hospital is considered one of the largest national centers because it serves a number of cities in Northern Egypt. We also included a large sample of 785 individuals, as we had recruited all PLWHA who met the inclusion criteria.

Additionally, we used a hospital pharmacy database to measure levels of adherence. Pharmacy registry may overestimate adherence, given that it assumes that all patients take their medication when they receive it. However, the interviewer was a former pharmacy staff member and was fully aware of the pattern of the adherence level and, thus, was able to build a degree of trust between her and the study participants.

Furthermore, because this is a cross-sectional study, we were unable to evaluate changes in stigma and discrimination over time.

Despite these limitations, the present study has many strengths. To the best of our knowledge, this is the first large-sized study to examine predictors of adherence to ART and stigma among PLWHA in Egypt. The published research on factors influencing adherence to ART is rather scarce in Egypt and the Middle East region. We believe that this research data will reflect the relevant data of a larger population, taking into consideration that Alexandria Fever Hospital is a pooling hospital that is one of the main centers hosting the National AIDS Program in Egypt.

## Data Availability

The data that support the findings of this study are available on request.
